# In Vitro Evaluation of Gastric Acid and Toothbrushing Effect on the Surface State of Different Types of Composite Resins

**DOI:** 10.3390/medicina58091281

**Published:** 2022-09-15

**Authors:** Ionuț Tărăboanță, Gabriela Gelețu, Simona Stoleriu, Gianina Iovan, Nicoleta Tofan, Andra Claudia Tărăboanță-Gamen, Andrei Georgescu, Cosmin Gabriel Popa, Sorin Andrian

**Affiliations:** Faculty of Dental Medicine, Grigore T. Popa University of Medicine and Pharmacy, 16 Universitatii Str., 700115 Iasi, Romania

**Keywords:** surface roughness, composite resin, toothbrush, hydrochloric acid, microhybrid, nanohybrid, profilometry

## Abstract

*Background and Objectives*: The aim of this in vitro study was to evaluate the effect of gastric acid associated with the effect of toothbrushing on the surface roughness of different types of composite resin used for direct restorations. *Materials and Methods*: The materials used in this study were two microhybrid (Filtek Z250, Herculite XRV) and two nanohybrid (Filtek Z550, Herculite XRV Ultra) composite resins. Two hundred and forty cylindrical samples with a height of 2 mm and a diameter of 6 mm were divided into four groups (groups A, B, C and D) corresponding to each tested material (n = 60). Each group was divided in two subgroups: subgroup I—the samples were submersed in hydrochloric acid and immediately submitted to toothbrushing; subgroup II—the samples were submitted only to toothbrushing. The simulation of the acid attack was performed by immersing the samples in a 0.01 M hydrochloric acid solution for 90 min. This procedure was followed immediately by toothbrushing simulation with 10,000 cycles. The acid attack and toothbrushing simulation were performed for two times. The surface roughness evaluation was performed with a Proscan 2100 profilometer. Repeated Measures ANOVA and Bonferroni *post-hoc* tests were used to perform the statistical analysis. *Results*: Simulation of one year of toothbrushing associated or not to hydrochloric acid exposure increases the surface roughness of microhybrid and nanohybrid composite resins. Six months of toothbrushing associated to six months of hydrochloric acid exposure increase the surface roughness of nanohybrid composite resins. *Conclusions*: Microhybrid composite resins surface becomes rougher after toothbrush and acid submersion when comparing to nanohybrid composite resins.

## 1. Introduction

Introduced by Bowen by the middle of the 20th century, composite resins have now become the first choice for dental practitioners. The wide use of these materials has attracted a great interest of researchers in the field, which has led to the development of a wide variety of composite resins. The chemical, physical and mechanical properties of composite resins are dependent to a number of factors such as the type of resin matrix, the type, distribution and size of filler particles, the types of coupling agents, photo-initiators or activators [1] and mechanical wear [2].

The main directions of the development the composite resins aimed to improve their filling technology by continuously changing the weight and the volume of loading, the size, the morphology or to incorporate new types of inorganic particles and to obtain their silanization [1]. In the technological development of composite resins, new types of resin monomers were also pursued. However, reducing the particle size and increasing their volume ratio have diminished the maneuverability and mechanical properties of the composite resins [1,3].

Although in dental therapy the restoration of Black class I and II cavities require the use of materials with increased mechanical properties, while the anterior restorations mostly require the use of materials with increased aesthetic properties [4]. A material that is able to meet all these goals has not yet been discovered [5]. Microhybrid composite resins were considered to be the most suitable materials in terms of maneuverability, strength and aesthetics [2]. This type of composite resin has inorganic particles with micronic (1–5 μm) and submicronic (0.4–0.8 μm) dimensions, as well as silicon particles with dimensions between 0.04–0.05 μm [6]. However, progresses in recent years have brought a new type of composite resin, with nanometric particles. These materials manage to combine the resistance of hybrid composite resins, with the polishability of those with micronic particles, have a much better wear resistance and a reduced polymerization shrinkage compared to other composite resins [6,7].

The term “nanotechnology” refers to “anything smaller than microtechnology”, such as the nanometric particles in nanohybrid composite resins [6,7]. The nanohybrid resins contain particles with dimensions of 0.4–5 microns [4]. One of the main advantages mentioned in terms of composite resins with nanometric particles, is their special aesthetics [3]. This property is favored by the inability of the human eye to distinguish particles of very small size [6,7,8].

For dental restorations the longevity, aesthetics and long-term clinical success are directly related to the surface roughness [4]. Thus, the smoother is the surface, the lower will be the accumulation of bacterial plaque or the discolorations [1,8]. The aesthetics of the restorations is also influenced by the surface roughness. A rough surface will not reflect the light in a proper way, thus affecting the aesthetic proprieties of the restoration [5]. The wear caused by food, intrinsic acids or oral hygiene products can negatively influence the surface characteristics of the restoration and their aesthetics [9,10].

The influence of oral hygiene products on the surface condition of restorations depends on their characteristics, such as the hardness of the toothbrush bristles, the RDA (Relative Dentin Abrasion), the pH or the amount of toothpaste used [2]. The nanotechnology cand be found in the composition of the toothpastes, as they contain calcium carbonate particles with nanometric dimensions [6]. Calcium carbonate is found in most commercial toothpastes and has both remineralizing and abrasive roles [6,11,12,13].

A number of studies have shown that toothbrushing tends to increase the surface roughness of composite resins [6,11,12]. In a study conducted by Oliviera et al., it was concluded that the surface roughness of nanohybrid composite resins after toothbrushing is lower than the roughness of the microhybrid resins due to the smaller particle size, which may favor a more homogeneous distribution of the particles in the matrix [13].

In addition to mechanical wear, composite resins can be degraded by the erosive action of the acids with which they come in contact [14]. Low pH values of extrinsic acids from food or beverages or intrinsic acids such as hydrochloric acid, can negatively influence the physical, chemical or mechanical characteristics of restorative materials [2]. The degradation of restorative materials can be a consequence of pH value variation in the oral cavity [14]. In the mechanism of acid action, not only the pH is of particular importance, but also the structural characteristics of acid molecules [10,14]. Very little data are available in the scientific literature regarding acid interactions with the surface of composite restorations [14,15].

The aim of the present study was to evaluate the surface roughness of two nanohybrid and two microhybrid composite resins after submersion in hydrochloric acid and after the abrasive action of toothbrushing. The null hypothesis was that there are no statistically significant differences between the surface roughness of the materials, after acid submersion and tooth brushing procedures.

## 2. Materials and Methods

### 2.1. Sample Preparation

The sample size was calculated using G * Power software (version 3.1.9.7., Heinrich-Heine-Universität Düsseldorf, Düsseldorf, Germany). The used effect size was 0.25 which is a medium effect in Cohen classification. The alpha value was 0.05 with a power of 95%. The results estimated a number of 27 samples required for each group.

**Figure 1:**
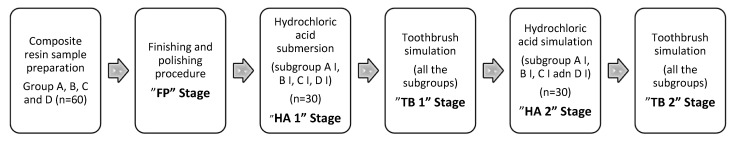
Study design.

Two microhybrid composite resins (Filtek Z250, 3M ESPE, St. Paul, MN, USA and Herculite XRV, Kerr, Orange, CA, USA) and two nanohybrid composite resins (Filtek Z550 3M ESPE, St. Paul, MN, USA and Herculite XRV Ultra, Kerr, Orange, CA, USA) were used in this study. The materials structure and composition are presented in Table 1. The study design is presented in Figure 1.

Sixty cylindrical samples of each tested material were prepared and included in four study groups: group A (Filtek Z250), group B (Filtek Z550), group C (Herculite XRV) and group D (Herculite XRV Ultra). Each study group was divided in two subgroups: subgroups I—the samples were submersed in hydrochloric acid and then submitted to toothbrushing procedure; subgroups II—the samples were submitted exclusively to toothbrushing.

The samples consisted in disks with a height of 2 mm and a diameter of 6 mm obtained by placing the materials in acrylic molds. The material was inserted in a single increment in the mold that was placed on a glass plate and covered with another glass plate. Between the material and the glass plates, a celluloid matrix was placed to create smooth surfaces. To remove the excess material and the air bubbles, a constant pressure using a weight of 500 g was applied for 30 s. The composite resin was then light-cured for 40 s through the glass plate, using a Bluephase 20i light-curing lamp (Ivoclar, Vivadent, Schaan, Liechtenstein) with a light intensity of 1200 mW/cm^2^, and a wavelength range from 385 to 515 nm. The light intensity was measured with a Bluephase Meter II radiometer (Ivoclar Vivadent, Schaan, Liechtenstein). Then the samples were removed from the mold and submersed in distilled water for 24 h.

### 2.2. Finishing and Polishing Procedure

Sof-Lex Finishing and Polishing System (Batch No. NC11346, 3M ESPE, St. Paul, MN, USA) was used to perform this step (“FP stage”). The system consists in two disposable wheels (beige and white), made of a thermoplastic elastomer impregnated with aluminum oxide particles. The beige wheel is indicated for finishing, smoothing and removal of scratches and the white one is indicated for polishing. Each spiral wheel was used only one time for 30 s for each sample, without water cooling or polishing paste. A contra-angle handpiece, at a speed of 20,000 rpm was used to activate the wheels.

### 2.3. Simulation of Acid Attack

Thirty samples from each group were submersed in a 0.01 M hydrochloric acid with a pH of 3.8 (subgroups A I, B I, C I, D I). The solution pH was verified with a pH-meter (Thermo Scientific Eutech pH 5+, Vernon Hills, IL, USA) before the submersion of the samples and by the end of the acid cycles. The hydrochloric acid solution was changed every 60 min in order to keep a constant pH value. The acid attack simulation was performed in two distinct cycles (stages “HA 1” and “HA 2”) of 90 min each, in an incubator (Biobase BJPXH30II, Biodusty, Shandong, China), at a constant temperature of 37 °C. The first acid cycle “HA1” took place immediately after finishing and polishing procedure and the second cycle “HA2” was performed after the first toothbrushing session “TB1”.

### 2.4. Brushing Simulation

Toothbrushing was performed using a brushing simulation device, in two distinct steps (“TB 1” and “TB 2”) of 5000 brushing cycles each, with an intensity of 100 cycles /minute and a constant load of 500 g. The first brushing procedure “TB1” was performed immediately after the first acidic attack “HA1” and the second brushing procedure “TB1” was realized immediately after the second submersion in hydrochloric acid “HA2”. Brushing was performed using a medium hardness bristle toothbrush (Toothbrush R.O.C.S. Professional Medium, Tallinn, Estonia) made of nylon, with a bristle length of 0.8/1.3 cm and a thickness of 1.8/2.0 mm and a toothpaste slurry, obtained by mixing a medium RDA (Relative Dentin Abrasivity) toothpaste (Prodent Cool Mint, Amersfoort, The Netherlands) and distilled water in 1:3 ratio. Then, the samples were rinsed under running water, dried for 2 min using the air spray from the dental unit and stored in deionized water for 24 h.

### 2.5. Profilometry

Surface roughness of the samples were assessed using profilometric measurements after finishing and polishing procedure, acidic challenge and toothbrush cycles. The mean (Ra) values of the surface roughness were recorded using a non-contact profilometer (Proscan 2100, Scantron Ltd., Taunton, Somerset, Great Britain). The used cut-off value was 0.4 mm with a navigating distance of 4 mm. The stylus tip had 5 μm and was activated with 4 mN force and 0.5 m/s speed. The measurements were performed for twenty times with crossing directions for each sample. Mean Ra values were obtained as a result of three distinct determinations, each sample being rotated with a 120° angle.

### 2.6. Statistical Analysis

The values recorded between and within the groups were analyzed using an IBM SPSS software package (version 26.0, SPSS Inc., Chicago, IL, USA). Parametric tests Repeated Measures ANOVA and *post-hoc* Bonferroni were used to settle the differences between the mean Ra values, with a significance level of *p* < 0.05.

## 3. Results

**Figure 2:**
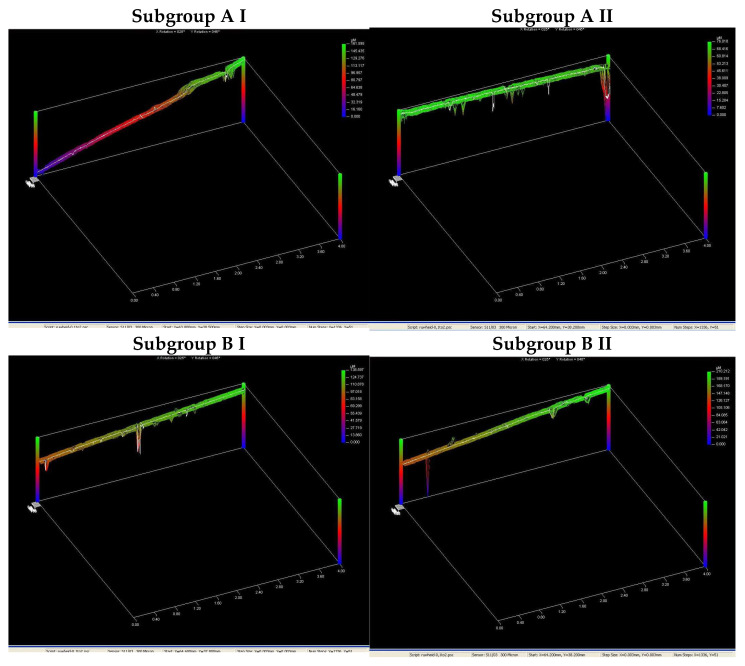

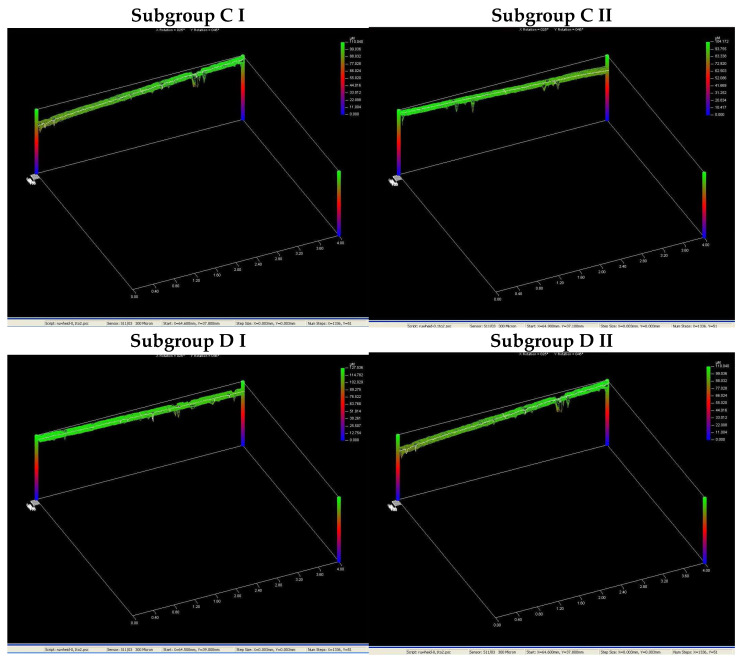
Profilometric measurements of some samples in each subgroup in stage TB2.

In Figure 2 are shown some of the profilometric measurements of the samples from each subgroup at the end of TB 2 stage.

**Figure 3:**
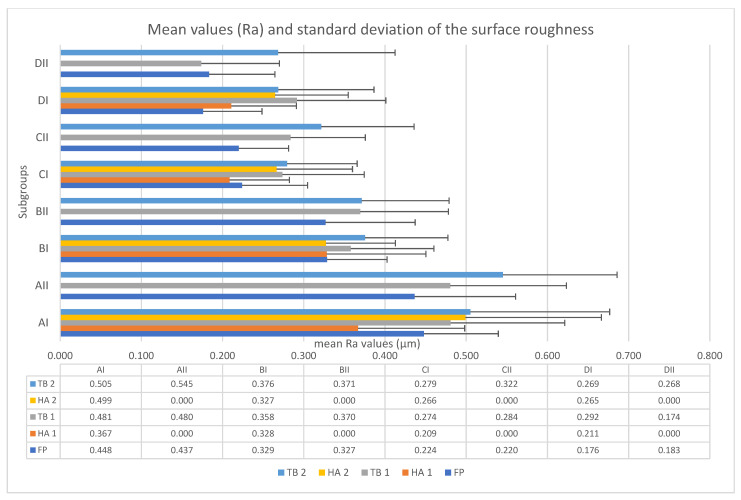
Boxplot representation of the mean (Ra) values **and standard deviation** of each subgroup in each stage.

The mean Ra values and standard deviation of the surface roughness of each subgroup in each stage is presented in the boxplot below (Figure 3).

In Table 2 are shown the statistical results of comparison the data from subgroups in stage TB 2. Significant differences were recorded when comparing the mean Ra values between subgroups A I and B I; A I and B II; A I and C I; A I and C II; A I and D I; A I and D II; A II and B I; A II and B II; A II and C I; A II and C II; A II and D I; A II and D II; B I and C I; B I and C II; B I and D I; B I and D II; B II and C II; B II and D I; B II and D II.

In Table 3 are shown the significant differences between the mean Ra values obtained in subgroups at the end of each stage. In subgroups A I, AII, B I, B II, C I, no significant differences were recorded. In subgroup C II, differences were recorded by the end of stage FP and TB 2. In subgroup D I, significant differences were recorded between the values in stages FP and TB 1. In subgroup D II, significant differences were found between the values obtained by the end of stages TB 1 and TB 2.

## 4. Discussion

Finishing and polishing procedures are very important steps to obtain an optimal restoration [16]. A number of studies have concluded that the surface roughness value of a restoration must be lower than 0.2 μm in order to avoid the adhesion and multiplication of the microorganisms [17,18].

The finishing and polishing procedure can be influenced by some factors such as the type of finishing system, the technique used, the type of the restorative material, the size of the filler particles or the type, the volume or the conversion degree of resin monomers [17].

In recent years, we assisted to the development of composite resins having inorganic particles of smaller dimensions which improved the surface polishability, boosted the mechanical properties and allowed their use for both anterior and posterior restorations [17,18]. In this study two types of microhybrid and two types of nanohybrid composite resins were used, whose organic matrices were based on BisGMA monomer [16,18].

For finishing and polishing procedure, two steps Sof-Lex system was used. According to previous studies, this finishing and polishing system had been considered to be the most efficient when comparing to other systems [18]. During finishing procedure the resin matrix is initially removed due to its lower hardness while the filling particles are exposed and subsequently dislocated from the resin matrix, thus increasing the surface roughness [19,20].

A series of studies which evaluated the surface roughness of composite resins after finishing and polishing procedure had concluded that the degree of material finishability depends on two factors: the size of the filling particles of the composite resin and the size of abrasive particles of the finishing system [19,20]. In this study, we tested microhybrid composite resins composed of zirconium/silicium and barium/silicium particles with dimensions between 0.01–3.5 µm and nanohybrid composite resins based on zirconium/silicium, barium glass fillers and silicon dioxide particles with dimensions between 20–50 nm. Regarding the type of composite filler, studies have found that the smoothest surface was recorded for barium-based particles [21]. An effective finishing and polishing system must contain abrasive particles with a higher hardness than the filler of restorative material, otherwise only the organic component of the composite resin will be abraded [22,23]. Sof-Lex finishing and polishing system consists of aluminum oxide discs with a very high efficiency [13,24].

In our study was simulated the effect of gastric acid associated to toothbrushing after finishing and polishing procedure on the surface condition of microhybrid and nanohybrid composite resins. The null hypothesis of the study was that there are no changings on surface roughness of the materials after the acid attack associated to toothbrushing and it was rejected. The research hypothesis was based primarily on previous studies that found that composite resin-based materials can undergo degradation processes that consist in the wear of the organic matrix of the material by dissolving it [25,26,27] and subsequently exposing the inorganic particles, as a result of subjection to acidic conditions [27,28,29].

The abrasiveness of the toothbrushes can negatively influence the connection between the organic matrix and the filling particles, determining the loss of organic matrix which results in the exposure of the inorganic particles [30,31]. Previous studies have shown that abrasion due to toothbrushing and acid erosion act synergistically in increasing the composite surface roughness [30,31,32,33].

In our study we observed that the samples brushed immediately after the finishing and polishing procedure recorded and increased surface roughness compared to the samples that were brushed immediately after the first acidic challenge for all the materials, except for Herculite XRV Ultra. Toothbrushing for one year increased the surface roughness of all the samples when compared to the samples that were exclusively finished and polished or submersed in hydrochloric acid. One year of toothbrushing associated to one year of hydrochloric acid action showed a decreased surface roughness compared to one year of toothbrushing without hydrochloric acid action for Hercultite XRV and Herculite XRV Ultra.

A number of studies had concluded that the wear resistance of composites was related to the type, size and degree of composite loading with inorganic particles [20,28]. In our study nanohybrid composite resins (Filtek Z550 and Herculite XRV Ultra) recorded lower surface roughness when comparing to the tested microhybrid composites Filtek Z250 and Herculite XRV after one year of toothbrushing and one year of hydrochloric acid action. Composite resins with small filler particles have a higher wear resistance [34,35]. For microhybrid composites, large fillers are lost due to wear, creating larger surface defects when comparing to nanohybrid ones [25,26,29]. Also, the lower filling volume of nanohybrid composites can favor an uniform dispersion of particles in the organic matrix and thus increasing the wear resistance [29,30,36]. In our study the tested microhybrid composite resins had filler weight between 79–82% and filler volume of 60% and the tested nanohybrid resins had filler weight between 78–82% and filler volume of 68%.

When comparing the behaviour of Filtek Z550 nanohybrid resin and Filtek Z250 microhybrid resin, we noticed that the surface of Filtek Z250 resin was much rougher than the surface of Filtek Z550, by the end of each stage. Microhybrid composite resin Herculite XRV showed an increased surface roughness comparing to nanohybrid composite resin Herculite XRV Ultra.

In the present study, the samples were submersed in a 0.01 M hydrochloric acid solution having a pH of 3.8 in two sessions of 90 min and then stored in deionized water at a constant temperature of 37 °C. According to a study conducted by Yehia et al., submersion in hydrochloric acid with 3.8 pH for 3 h is equivalent to 1 year of gastric juice exposure [37]. Although the gastric acid pH varies between 1.4 and 1.6, in our study we have chosen the diluted acid considering that in the oral environment the acidity is slightly buffered by saliva [29,31,38,39,40]. In addition, the literature mentions that saliva neutralizes acids on dental surfaces in about 3 min [33,34]. For maintaining the H^+^ ions values as constant as possible, the acid solution was changed every hour.

Regarding toothbrushing simulation, previous studies have established that the brush comes into contact with the tooth or restorative material for 10 s during each brushing session, so in our study the two toothbrushing simulation cycles with a total time of 100 min would be the equivalent of 1 year of toothbrushing [32,39,41]. The simulation of toothbrushing was performed using a device consisting of 10 toothbrushes with medium hardness bristles, electrically operated, with a frequency of 100 cycles per minute.

The conclusions of this in vitro study are limited by the small number of methods to which the tested materials were subjected. Future in vitro studies are needed to replicate the oral environment by simulating the salivary flow, the enzyme activity, the microbial action, the aging and thermocycling processes or the chewing cycles.

## 5. Conclusions

One year of toothbrushing and one year of toothbrushing associated to hydrochloric acid submersion increase the surface roughness of microhybrid and nanohybrid composite resins. Six months of toothbrushing associated to six months of hydrochloric acid exposure increase the surface roughness of microhybrid and nanohybrid composite resins. Microhybrid composite resins surface becomes rougher after exposing to hydrochloric acid and toothbrushing for six months and one year when comparing to nanohybrid composite resins. These results offer new perspectives regarding the use of microhybrid and nanohybrid composite resins as materials for direct restorations in patients with gastroesophageal reflux.

## Figures and Tables

**Table 1:** Composition and structure of the materials used in study.

Composite Resin	Manufacturer	Organic Matrix	Filler Type/Dimension; % wt/vol	Batch No.
Filtek Z250	3M ESPE, St. Paul, MN, USA	BisGMA, UDMA, BisEMA	Zirconium/silicium; microhybrid; 82 wt%/60 vol% 0.01–3.5 µm	NA55912
Filtek Z550	3M ESPE, St. Paul, MN, USA	BisGMA, UDMA, BisEMA, TEGMA, PEGDMA	Zirconium/silicium; nanohybrid; 82 wt%/68 vol% Non-agglomerated/non-aggregated particles 20 nm Clusters of aggregated particles 3 µm	N991254
Herculite XRV	Kerr corporation, Orange, CA, USA	BisGMA, TEGDMA, UDMA	Barium/silicium 79 wt%; microhybrid 0.6 µm	7170484
Herculite XRV ultra	Kerr corporation, Orange, CA, USA	BisGMA, TEGDMA	Barium glass fillers; silicon dioxide; Submicronic particles (0.4 microns); nanosized particles (50 nm); prepolymerized particles (25 µm); 78 wt%; nanohybrid	7407915

Bis-GMA—Bisphenol A diglycidyl ether methacrylate; Bis-EMA—Bisphenol-A ethoxylated dimethacrylate; TEGDMA—Triethylenglycol dimethacrylate; UDMA—Urethane dimethacrylate; HEMA—Hydroxyethyl methacrylate; PEGDMA—poly(ethylene glycol) dimethacrylate.

**Table 2:** Comparison of the data between the subgroups of each group in stage TB2.

Groups/Subgroups		A		B		C		D	
		A I	A II	B I	B II	C I	C II	D I	D II
**A**	A I	-	*	** 0.011	** 0.009	** 0.000	** 0.000	** 0.000	** 0.000
	A II	*	-	** 0.037	** 0.030	** 0.000	** 0.000	** 0.000	** 0.000
**B**	B I	** 0.011	** 0.037	-	*	** 0.049	** 0.033	** 0.000	** 0.001
	B II	** 0.009	** 0.030	*	-	*	** 0.040	** 0.000	** 0.001
**C**	C I	** 0.000	** 0.000	** 0.049	*	-	*	*	*
	C II	** 0.000	** 0.000	** 0.030	** 0.040	*	-	*	*
**D**	D I	** 0.000	** 0.000	** 0.000	** 0.000	*	*	-	*
	D II	** 0.000	** 0.000	** 0.001	** 0.001	*	*	*	-

- No values. * Statistically not significant. ** Statistically significant (*p* < 0.05).

**Table 3:** Differences between the subgroups in each stage.

**A I**						**A II**					
Stage	FP	HA1	TB1	HA2	TB2	Stage	FP	HA1	TB1	HA2	TB2
FP	-	*	*	*	*	FP	-	-	*	-	*
HA1	*	-	*	*	*	HA1	-	-	-	-	-
TB1	*	*	-	*	*	TB1	*	-	-	-	*
HA2	*	*	*	-	*	HA2	-	-	-	-	-
TB2	*	*	*	*	-	TB2	*	-	*	-	-
**B I**						**B II**					
Stage	FP	HA1	TB1	HA2	TB2	Stage	FP	HA1	TB1	HA2	TB2
FP	-	*	*	*	*	FP	-	-	*	-	*
HA1	*	-	*	*	*	HA1	-	-	-	-	-
TB1	*	*	-	*	*	TB1	*	-	-	-	*
HA2	*	*	*	-	*	HA2	-	-	-	-	-
TB2	*	*	*	*	-	TB2	*	-	*	-	-
**C I**						**C II**					
Stage	FP	HA1	TB1	HA2	TB2	Stage	FP	HA1	TB1	HA2	TB2
FP	-	*	*	*	*	FP	-	-	*	-	** 0.002
HA1	*	-	*	*	*	HA1	-	-	-	-	-
TB1	*	*	-	*	*	TB1	*	-	-	-	*
HA2	*	*	*	-	*	HA2	-	-	-	-	-
TB2	*	*	*	*	-	TB2	** 0.002	-	*	-	-
**D I**						**D II**					
Stage	FP	HA1	TB1	HA2	TB2	Stage	FP	HA1	TB1	HA2	TB2
FP	-	*	** 0.015	*	*	FP	-	-	*	-	*
HA1	*	-	*	*	*	HA1	-	-	-	-	-
TB1	** 0.015	*	-	*	*	TB1	*	-	-	-	** 0.034
HA2	*	*	*	-	*	HA2	-	-	-	-	-
TB2	*	*	*	*	-	TB2	*	-	** 0.034	-	-

- No values. * Statistically not significant. ** Statistically significant (*p* < 0.05).
